# Clinical Notes upon Two Years' Surgical Work in the Liverpool Royal Infirmary

**Published:** 1884-12

**Authors:** 


*Clinical Notes upon two years' Surgical Work in the Liverpool Royal Infirmary*. By W. MITCHELL BANKS. Pp. 190. Printed for the Author. 1884.

Not all the best writing is to be found in elaborate treatises, and not always is most knowledge gained from those who make greatest pretence of instructing us. Our moral is pointed by a perusal of the charming little work before us. The author has taken us into his confidence, and, *currente calamo*, has gossiped over his past two years' work at the Liverpool Infirmary, and shared with us the experiences of his surgical lifetime. One thought forces itself upon us as we lay down the book; it is this: With what a manly grasp of strong common-sense does the practical provincial English surgeon pick out the possibilities of every theoretical advance in the practice of surgery, ignoring alike the juvenile twaddle of continental thesis-writers and the fame-compelling inventions of enthusiastic embryos in our British metropolis. Whether we believe in all that the writer says or not, every idea comes home to us with a feeling of genuineness which is as novel as it is pleasing. Our pushing writers nowadays have got into a way of editing other people's ideas, and quoting their authors' names in polyglot at the bottoms of their pages; Mr. Banks speaks out for himself from his own lights, and is clear, original, and instructive accordingly.

